# Preoperative hypokalemia can increase complications after colorectal cancer surgery: a propensity score matching analysis

**DOI:** 10.1186/s12885-022-09950-1

**Published:** 2022-08-03

**Authors:** Bin Zhang, Xiao-Yu Liu, Bing Kang, Chao Yuan, Zi-Wei Li, Zheng-Qiang Wei, Dong Peng

**Affiliations:** 1 Department of Gastrointestinal Surgery, the First Affiliated Hospital of Chongqing Medical University, Chongqing, 400016 China; 2 Department of Clinical Nutrition, The First Affiliated Hospital of Chongqing Medical University, Chongqing, 400016 China

**Keywords:** Colorectal cancer, Short-term outcomes, Hypokalemia, Propensity score matching, Surgery

## Abstract

**Background:**

Whether hypokalemia can affect the short-term outcomes of CRC patients after radical surgery remains unclear. The purpose of this study was to investigate the impact of preoperative hypokalemia on the short-term outcomes for colorectal cancer (CRC) patients who underwent radical CRC surgery using propensity score matching (PSM).

**Methods:**

We retrospectively enrolled consecutive CRC patients from Jan 2011 to Dec 2021 in a single-center hospital. Hypokalemia was defined as a serum potassium concentration < 3.5 mmol/L. The short-term outcomes were compared between the hypokalemia group and the normal blood potassium group. In addition, univariate and multivariate logistic regression analyses were conducted to identify independent risk factors for overall complications.

**Results:**

A total of 6183 CRC patients who underwent radical surgery were included in this study, of whom 390 (6.3%) patients were diagnosed with hypokalemia before surgery. After 1:1 ratio PSM, there were 390 patients in the hypokalemia group and in the normal potassium group. No significant difference was found between the two groups after PSM in terms of baseline information (*p* > 0.05). Regarding short-term outcomes, the hypokalemia group had a longer hospital stay (*p* = 0.028), a higher proportion of overall complications (*p* = 0.048) and a higher incidence of postoperative pneumonia (*p* = 0.008) after PSM. Moreover, hypokalemia (*p* = 0.036, OR = 1.291, 95% CI = 1.017–1.639) was an independent risk factor for overall complications.

**Conclusion:**

Preoperative hypokalemia could increase complications after CRC surgery and prolong the hospital stay. Moreover, preoperative hypokalemia was an independent risk factor for overall complications.

## Introduction

Colorectal cancer (CRC) is the third most common cancer in the world and the second leading cause of cancer-related death [1]. It has been reported that the incidence of CRC will double by 2035 globally [2]. Surgery is the core treatment for CRC patients [3–5], and favorable short-term outcomes can reduce the mental and physical stress of patients as well as lighten their financial burden simultaneously [6, 7]. Thus, the short-term outcomes of CRC patients after radical surgery are major concerns to surgeons [8]. As reported previously, the short-term outcomes after CRC surgery are affected by many factors, including age [9], comorbidities such as liver cirrhosis [10] and diabetes [11], surgical approaches [12] and operation time [13, 14].

Hypokalemia is a type of electrolyte disturbance that can result in myasthenia, enteroparalysis and even severe life-threatening arrhythmia [15, 16]. For CRC patients, preoperative hypokalemia can be caused by bowel cleansing preparation, ileus, and inadequate intake of potassium [17, 18]. Previous studies demonstrated that preoperative hypokalemia could lead to adverse consequences in patients after noncardiac surgery and open abdominal surgery [19, 20]; however, it remains unclear whether hypokalemia could affect short-term outcomes especially for CRC patients after radical surgery.

Therefore, the purpose of our study was to investigate the impact of preoperative hypokalemia on the short-term outcomes of CRC patients who underwent radical CRC surgery.

## Methods

### Patients

We retrospectively collected CRC patients after radical surgery from Jan 2011 to Dec 2021 in a single-center hospital. This study was processed according to the World Medical Association Declaration of Helsinki. Ethical approval from the institutional review board of the First Affiliated Hospital of Chongqing Medical University was obtained (2021–536) and all patients signed informed consent forms.

### Inclusion and exclusion criteria

**Fig. 1:**
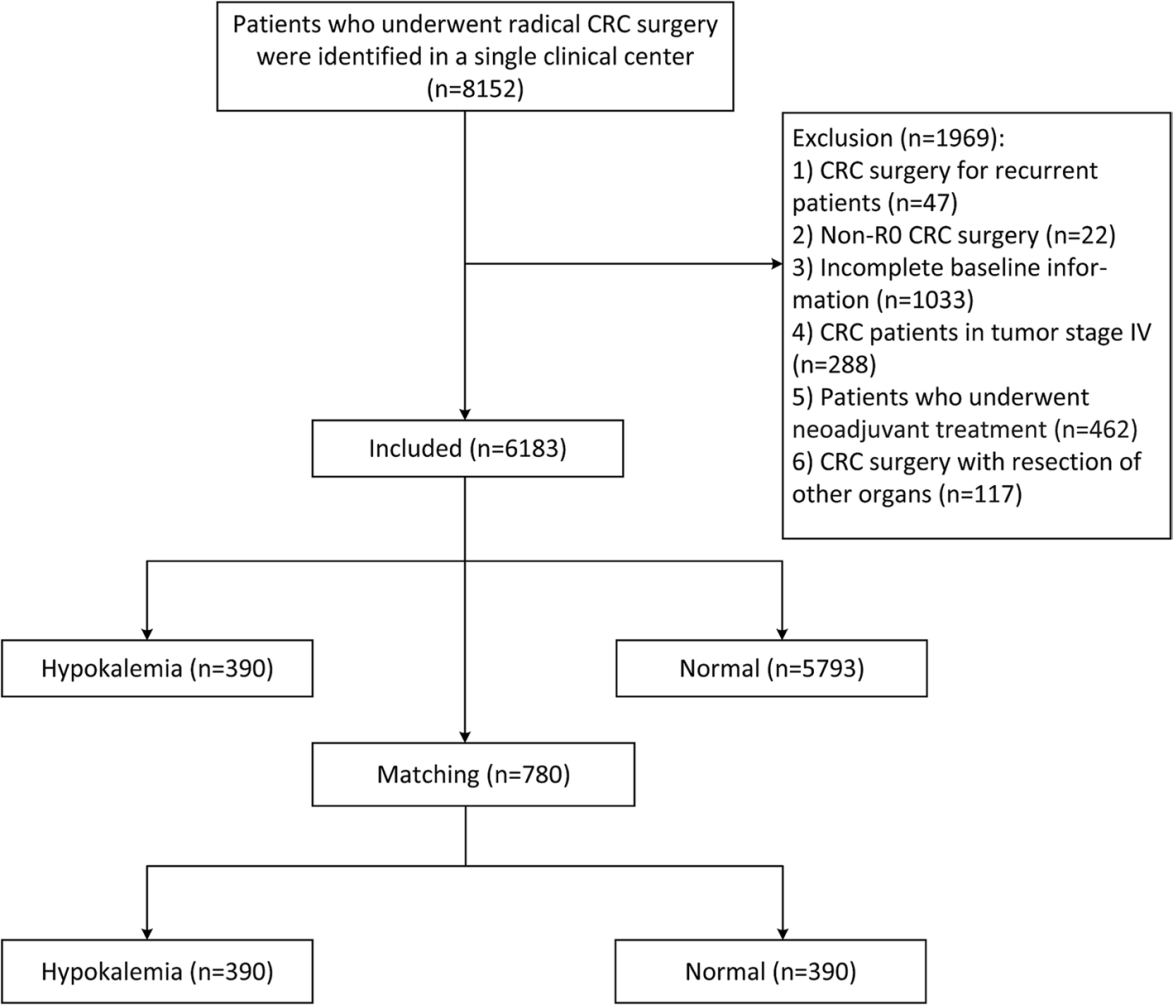
Flow chart of patient selection

We identified 8152 CRC patients who underwent radical CRC surgery from a single center hospital. The exclusion criteria were as follows: 1, CRC surgery for recurrent patients (*n* = 47); 2, non-R0 CRC surgery after pathology confirming (*n* = 22); 3, incomplete baseline information in the medical system (*n* = 1033); 4, CRC patients in tumor stage IV (*n* = 288); 5, patients who underwent neoadjuvant treatment (*n* = 462) and 6, CRC surgery with resection of other organs (*n* = 117). Finally, a total of 6183 patients were included in this study (Fig. 1).

### The management of hypokalemia

The value of serum potassium was identified by the first blood test after admission. Intravenous potassium supplementation was implemented if hypokalemia was identified and we re-examined serum potassium on the day before surgery or the surgery day to ensure that patients were eligible for general anesthesia and surgery.

### Surgery management

All the patients included in this study underwent elective surgery, and bowel preparation with oral laxatives or enemas was carried out on the day before surgery. Moreover, fasting for 8 h before surgery was required for all patients. All patients underwent radical resection according to the clinical guidelines and total mesorectal excision or complete mesocolic excision was performed. The pathology confirmed R0 resection.

### Definitions

We defined hypokalemia as a serum potassium concentration < 3.5 mmol/L. Serum potassium ranging from 3.0 to 3.5 mmol/L, 2.5 to 3.0 mmol/L and < 2.5 mmol/L was defined as slight, moderate, and severe hypokalemia, respectively. The tumor node metastasis (TNM) stage was diagnosed according to the AJCC 8^th^ Edition [21]. The complications were defined according to the Clavien-Dindo classification [22].

### Data collection

The medical information of the enrolled patients was collected from the outpatient and inpatient systems. The baseline information was gathered, including serum potassium concentration, age, sex, body mass index (BMI), smoking, drinking, hypertension, type 2 diabetes mellitus (T2DM), coronary heart disease (CHD), surgical history, surgical methods, tumor size, tumor location and tumor stage. The short-term outcomes, including operation time, blood loss, hospital stay and overall complications, were collected. Complications including anastomotic leakage, incision infection, pneumonia, lymph fistula, ileus, venous thromboembolism (VTE), reoperation, postoperative death and other complications were recorded.

### Propensity score matching (PSM)

We conducted PSM between the hypokalemia group and the normal potassium group. Nearest neighbor matching was performed without replacement at a 1:1 ratio and a caliper width with a 0.01 standard deviation was specified. The baseline information, including age, sex, BMI, smoking, drinking, hypertension, T2DM, CHD, surgical history, surgical methods, tumor size, tumor location and tumor stage, was matched. The standardized mean difference for all the matching variables ranged from 0.5 to 8.8% after PSM, which indicated a good performance of PSM.

### Statistical analysis

Continuous variables are expressed as the mean ± standard deviation (SD) and an independent-sample t-test was adopted to compare the difference between the hypokalemia group and the normal potassium group. Categorical variables are expressed as absolute values and percentages, and the chi-square test or Fisher’s exact test was used. Univariate and multivariate logistic regression analyses were conducted to identify independent predictive factors for overall complications. Data were analyzed using SPSS (version 22.0) statistical software and R software version 2.10.1. A bilateral *p* value of < 0.05 was considered statistically significant.

## Results

### Patients

A total of 6183 CRC patients who underwent radical surgery were included in this study according to the inclusion and exclusion criteria. Table 1 shows the clinical characteristics of the included patients. Among them, 390 (6.3%) patients were diagnosed with hypokalemia after admission in which 40 (0.65%) patients had moderate or severe hypokalemia, and others were at the normal level of serum potassium concentration. We conducted PSM between the hypokalemia group and the normal potassium group. After 1:1 ratio PSM, there were 390 patients in the hypokalemia group and in the normal potassium group, respectively (Fig. 1).

**Table 1:** Clinical characteristics of CRC patients

Characteristics	No. 6183
Age, year	63.2 ± 12.3
Sex	
Male	3598 (58.2%)
Female	2585 (41.8%)
BMI, kg/m^2^	22.6 ± 3.2
Smoking	2291 (37.1%)
Drinking	1876 (30.3%)
Hypertension	1586 (25.7%)
T2DM	679 (11.0%)
CHD	286 (4.6%)
Surgery history	1499 (24.2%)
Laparoscopy	5362 (86.7%)
K^+^	4.1 ± 0.4
Hypokalemia	390 (6.3%)
Tumor location	
Colon	2982 (48.2%)
Rectum	3201 (51.8%)
Tumor size	
< 5 cm	4247 (60.2%)
≥ 5 cm	2803 (39.8%)
TNM stage	
I	1197 (19.4%)
II	2598 (42.0%)
III	2388 (38.6%)
Blood loss, mL	97.5 ± 144.6
Operation time, min	220.8 ± 79.4
Hospital stay, day	11.3 ± 8.1
Overall complications	1339 (21.7%)

*Note*: Variables are expressed as the mean ± SD, n (%)

*Abbreviations*: *T2DM* Type 2 diabetes mellitus, *BMI* Body mass index, *CHD* Coronary heart disease

### Baseline characteristics of included patients before and after PSM

The baseline characteristics of the hypokalemia group and the normal potassium group were compared before and after PSM. The hypokalemia group had older age (*p* < 0.01), a higher proportion of females (*p* < 0.01), hypertension (*p* < 0.01), T2DM (*p* < 0.01), CHD (*p* = 0.003) and colon cancer (*p* = 0.001), and a lower proportion of drinking (*p* < 0.01), smoking (*p* < 0.01), laparoscopy (*p* < 0.01) and tumor size < 5 cm (*p* = 0.047) before PSM. However, no difference was found between the two groups after PSM in terms of baseline information (Table 2).

**Table 2:** Baseline characteristics before and after PSM

Characteristics	Before PSM				After PSM			
	Hypokalemia (390)	Normal (5793)	*P* value	SMD (%)	Hypokalemia (390)	Normal (390)	*P* value	SMD (%)
K^+^	3.2 ± 0.2	4.1 ± 0.4	< 0.01*	—	3.2 ± 0.2	4.1 ± 0.4	< 0.01*	—
Age (year)	66.9 ± 12.0	63.0 ± 12.2	< 0.01*	30.4	66.9 ± 12.0	66.7 ± 10.7	0.756	2.2
Sex			< 0.01*	32.7			0.217	8.8
Male	172 (44.1%)	3426 (59.1%)			172 (44.1%)	155 (39.7%)		
Female	218 (55.9%)	2367 (40.9%)			218 (55.9%)	235 (60.3%)		
BMI (kg/m^2^)	22.5 ± 3.3	22.6 ± 3.2	0.538	3.2	22.5 ± 3.3	22.6 ± 3.3	0.649	3.3
Smoking	107 (27.4%)	2184 (37.7%)	< 0.01*	22.0	107 (27.4%)	98 (25.1%)	0.464	5.2
Drinking	79 (20.3%)	1797 (31.0%)	< 0.01*	24.8	79 (20.3%)	74 (19.0%)	0.652	3.2
Hypertension	167 (42.8%)	1419 (24.5%)	< 0.01*	39.5	167 (42.8%)	157 (40.3%)	0.467	5.2
T2DM	67 (17.2%)	612 (10.6%)	< 0.01*	19.2	67 (17.2%)	59 (15.1%)	0.436	5.6
CHD	30 (7.7%)	256 (4.4%)	0.003*	13.7	30 (7.7%)	25 (6.4%)	0.484	5.0
Surgical history	95 (24.4%)	1404 (24.2%)	0.956	0.3	95 (24.4%)	102 (26.2%)	0.564	4.1
Laparoscopy	310 (79.5%)	5052 (87.2%)	< 0.01*	20.8	310 (79.5%)	317 (81.3%)	0.528	4.5
Tumor size			0.047*	10.3			0.943	0.5
< 5 cm	216 (55.4%)	3503 (60.5%)			216 (55.4%)	215 (55.1%)		
≥ 5 cm	174 (44.6%)	2290 (39.5%)			174 (44.6%)	175 (50.2%)		
Tumor location			0.001*	17.0			0.613	3.6
Colon	219 (56.2%)	2763 (47.7%)			219 (56.2%)	226 (57.9%)		
Rectum	171 (43.8%)	3030 (52.3%)			171 (43.8%)	164 (42.1%)		
Tumor stage			0.197	8.3			0.583	2.2
I	62 (15.9%)	1135 (19.6%)			62 (15.9%)	62 (15.9%)		
II	169 (43.3%)	2429 (41.9%)			169 (43.3%)	163 (41.8%)		
III	159 (40.8%)	2229 (38.5%)			159 (40.8%)	165 (42.3%)		

*Note*: Variables are expressed as the mean ± SD, n (%), *P-value < 0.05

*Abbreviations*: *PSM* Propensity score matching, *SMD* standardized mean difference, *T2DM* Type 2 diabetes mellitus, *BMI* Body mass index, *CHD* Coronary heart disease

### Short-term outcomes before and after PSM

Before PSM, the hypokalemia group had more intraoperative blood loss (*p* = 0.044), a longer hospital stay (*p* < 0.01), a higher proportion of overall complications (*p* = 0.001), a higher incidence of postoperative pneumonia (*p* < 0.01) and more postoperative death (*p* < 0.01) than the normal potassium group. After PSM, the hypokalemia group had a longer hospital stay (*p* = 0.028), a higher proportion of overall complications (*p* = 0.048) and a higher incidence of postoperative pneumonia (*p* = 0.008) as well (Table 3).

**Table 3:** Short-term outcomes before and after PSM

Characteristics	Before PSM			After PSM		
	Hypokalemia (390)	Normal (5793)	*P* value	Hypokalemia (390)	Normal (390)	*P* value
Operation time (min)	217.7 ± 79.3	221.0 ± 79.4	0.432	217.7 ± 79.3	217.8 ± 87.0	0.985
Blood loss (mL)	111.7 ± 273.3	96.5 ± 131.5	0.044*	111.7 ± 273.3	91.0 ± 96.3	0.158
Hospital stay (day)	12.8 ± 12.3	11.2 ± 7.7	< 0.01*	12.8 ± 12.3	11.2 ± 6.2	0.028*
Overall complications	111 (28.5%)	1228 (21.2%)	0.001*	111 (27.8%)	87 (22.3%)	0.048*
Anastomotic leakage	12 (3.1%)	144 (2.5%)	0.471	111 (28.5%)	5 (1.3%)	0.086
Incision infection	17 (4.4%)	198 (3.4%)	0.326	12 (3.1%)	12 (3.1%)	0.344
Pneumonia	27 (6.9%)	187 (3.2%)	< 0.01*	17 (4.4%)	11 (2.8%)	0.008*
Lymph fistula	6 (1.5%)	37 (0.6%)	0.051	27 (6.9%)	4 (1.0%)	0.524
Ileus	5 (1.3%)	117 (2.0%)	0.311	6 (1.5%)	10 (2.7%)	0.192
VTE	6 (1.5%)	52 (0.9%)	0.197	5 (1.3%)	2 (0.5%)	0.155
Reoperation	10 (2.3%)	95 (1.6%)	0.172	6 (1.5%)	3 (0.8%)	0.050
Postoperative death	7 (1.8%)	16 (0.3%)	< 0.01*	10 (2.3%)	3 (0.8%)	0.203
Other complications	45 (11.5%)	565 (9.8%)	0.252	7 (1.8%)	46 (11.8%)	0.911

*Note*: Variables are expressed as the mean ± SD, n (%), *P-value < 0.05

*Abbreviations*: *PSM* Propensity score matching, *VTE* Venous thromboembolism

### Univariate and multivariate logistic regression of the overall complications

Given that the overall complications were significantly different between the hypokalemia group and the normal potassium group, we conducted univariate and multivariate logistic regression to identify whether hypokalemia was an independent risk factor for overall complications in the whole cohort.

In univariate analysis, hypokalemia (*p* = 0.001, OR = 1.479, 95% CI = 1.177–1.859), age (*p* < 0.01, OR = 1.025, 95% CI = 1.020–1.031), open surgery (*p* < 0.01, OR = 2.275, 95% CI = 1.942–2.664), hypertension (*p* < 0.01, OR = 1.320, 95% CI = 1.154–1.509), T2DM (*p* < 0.01, OR = 1.473, 95% CI = 1.230–1.763), surgical history (*p* < 0.01, OR = 1.281, 95% CI = 1.117–1.469), smoking (*p* < 0.01, OR = 1.257, 95% CI = 1.111–1.423), drinking (*p* = 0.046, OR = 1.141, 95% CI = 1.002–1.300), CHD (*p* < 0.01, OR = 1.622, 95% CI = 1.251–2.103), tumor size (*p* < 0.01, OR = 1.273, 95% CI = 1.126–1.439) and intraoperative blood loss (*p* < 0.01, OR = 1.002, 95% CI = 1.002–1.003) were potential risk factors for overall complications. Moreover, hypokalemia (*p* = 0.036, OR = 1.291, 95% CI = 1.017–1.639), age (*p* < 0.01, OR = 1.020, 95% CI = 1.014–1.026), open surgery (*p* < 0.01, OR = 1.814, 95% CI = 1.533–2.146), T2DM (*p* = 0.01, OR = 1.290, 95% CI = 1.064–1.563), surgical history (*p* = 0.004, OR = 1.235, 95% CI = 1.071–1.425), smoking (*p* = 0.025, OR = 1.227, 95% CI = 1.026–1.468) and intraoperative blood loss (*p* < 0.01, OR = 1.002, 95% CI = 1.001–1.002) were independent predictors for overall complications in multivariate logistic regression analysis (Table 4).

**Table 4:** Univariate and multivariate logistic regression of the overall complications of the whole cohort

Risk factors	Univariate analysis		Multivariate analysis	
	OR (95% CI)	*P* value	OR (95% CI)	*P* value
Age, year	1.025 (1.020–1.031)	< 0.01*	1.020 (1.014–1.026)	< 0.01*
Surgical methods (open/laparoscopic)	2.275 (1.942–2.664)	< 0.01*	1.814 (1.533–2.146)	< 0.01*
Sex (male/female)	0.801 (0.708–0.908)	< 0.01*	0.873 (0.741–1.029)	0.105
BMI, Kg/m^2^	0.980 (0.962–0.999)	0.038*	0.987 (0.967–1.007)	0.189
Hypertension (yes/no)	1.320 (1.154–1.509)	< 0.01*	1.087 (0.936–1.263)	0.274
T2DM (yes/no)	1.473 (1.230–1.763)	< 0.01*	1.290 (1.064–1.563)	0.010*
Surgical history (yes/no)	1.281 (1.117–1.469)	< 0.01*	1.235 (1.071–1.425)	0.004*
Tumor location (colon/ rectum)	0.906 (0.803–1.023)	0.111		
Tumor stage (III/II/I)	1.013 (0.933–1.099)	0.767		
Smoking (yes/no)	1.257 (1.111–1.423)	< 0.01*	1.227 (1.026–1.468)	0.025*
Drinking (yes/no)	1.141 (1.002–1.300)	0.046*	0.965 (0.812–1.148)	0.690
CHD (yes/no)	1.622 (1.251–2.103)	< 0.01*	1.283 (0.974–1.689)	0.076
Tumor size (≥ 5/ < 5), cm	1.273 (1.126–1.439)	< 0.01*	1.120 (0.986–1.271)	0.081
K^+^ (Hypokalemia/ normal), g/L	1.479 (1.177–1.859)	0.001*	1.291 (1.017–1.639)	0.036*
Blood loss, mL	1.002 (1.002–1.003)	< 0.01*	1.002 (1.001–1.002)	< 0.01*

*Abbreviations*: *OR* Odds ratio, *CI* Confidence interval, *BMI* Body mass index, *T2DM* Type 2 diabetes mellitus, *CHD* Coronary heart disease

*P-value < 0.05

## Discussion

In this retrospective study, we enrolled a total of 6183 CRC patients who underwent radical surgery, of whom 390 patients were diagnosed with hypokalemia before surgery. After PSM, the hypokalemia group had a longer hospital stay and a higher proportion of overall complications, especially pneumonia. Furthermore, preoperative hypokalemia was an independent predictor for overall complications.

Some studies reported that preoperative hypokalemia was associated with adverse surgical short-term outcomes. Arora et al. reported that preoperative hypokalemia was an independent risk factor for the 30-day incidence of major adverse cardiovascular events (MACEs) and mortality after noncardiac surgery [19]. Similarly, a retrospective study reported that hypokalemia before surgery was accountable for higher a 30-day mortality after open abdominal surgery [20]. Nevertheless, these studies failed to analyze the impacts of preoperative hypokalemia especially in CRC patients. Although Zhu et al. reported that hypokalemia could prolong the first time to feces for patients undergoing laparoscopic colorectal resection [23], which might lengthen the hospital stay accordingly. However, the sample size was small, and the bias of confounding variables was not eliminated. Therefore, the impact of preoperative hypokalemia on the surgical complications for CRC patients remains unclear.

More than twenty percent of inpatients were diagnosed with hypokalemia [24]. Previous studies found that CRC patients were more likely to suffer from hypokalemia, which might partly be attributed to preoperative gastrointestinal preparation [17, 18, 23]. Therefore, focusing on the impact of preoperative hypokalemia on postoperative short-term outcomes for CRC patients is of great importance. We conducted this retrospective study with a relatively large sample size using PSM. In our research, the hypokalemia group had a significantly longer hospital stay and a higher proportion of overall complications than the normal potassium group.

The imbalance of serum potassium interferes with the cell membrane electrical potential [25, 26]. Hypokalemia reduced gastrointestinal motility and lead to delayed gastrointestinal function recovery after surgery [23]. Moreover, severe hypokalemia increased the risk of cardiac arrhythmias [27]. In this study, we also found that the hypokalemia group had a higher incidence of postoperative pneumonia. The underlying mechanism might due to hypokalemia causing fatigue and even myasthenia, and with the influence of abdominal pain, CRC patients who underwent surgery might have impaired respiration. This could lead to a higher incidence of lung infection [28]. In our study, although hypokalemia was corrected before surgery for all patients, the electrolyte status might be unstable. Thus, the impact of preoperative hypokalemia on gastrointestinal and respiratory function might not be completely eliminated after surgery. In addition, some studies reported that perioperative hypokalemia was a risk factor for postoperative hypokalemia [29, 30]. Taken together, these findings suggest that hypokalemia might increase the overall complications and prolong the hospital stay. More mechanisms need to be further investigated. Moreover, hypokalemia was more difficult to correct in the postoperative period than in the preoperative period [31], which indicates that surgeons should identify hypokalemia early before surgery.

Based on multivariate logistic regression, preoperative hypokalemia, age, open surgery, T2DM, surgical history, smoking and intraoperative blood loss were independent predictors of overall complications. To the best of our knowledge, preoperative hypokalemia had not been previously identified as an independent parameter of overall complications for CRC patients after radical surgery. However, we did not classify hypokalemia into different degrees according to serum potassium concentrations because only 40 patients were diagnosed with moderate or severe hypokalemia. Therefore, further studies are needed to investigate the influence of hypokalemia on specific complications.

To our knowledge, this was the first study to investigate the impact of preoperative hypokalemia on the short-term outcomes of CRC patients who underwent radical surgery. A total of 6183 CRC patients were enrolled in this study, which is a relatively large sample size. In addition, we adopted PSM to eliminate the bias of confounding factors, making the conclusion more reliable.

Some limitations existed in our study. First, this was a retrospective study conducted in a single clinical center; thus, selection bias was unavoidable despite the adoption PSM. Second, due to the lack of long-term follow-up, whether hypokalemia had a further impact on the long-term prognosis was unclear. Finally, the mechanisms by which preoperative hypokalemia increases postoperative complications are not comprehensive and need to be further studied. As a result, multicenter studies with large sample sizes should be performed to identify the correlation between hypokalemia and long-term prognosis in the future.

## Conclusions

This study demonstrated that preoperative hypokalemia could increase complications after CRC surgery and prolong the hospital stay. Moreover, preoperative hypokalemia was an independent risk factor for overall complications. Surgeons should attach more importance to the early identification of hypokalemia before surgery.

## Data Availability

The datasets used and analyzed during the current study are available from the corresponding author on reasonable request.

## Abbreviations

CRC: Colorectal cancer
PSM: Propensity score matching
TNM: Tumor node metastasis
BMI: Body mass index
T2DM: Type 2 diabetes mellitus
CHD: Coronary heart disease
